# Coronary Artery to Right Atrium Fistula Associated With First Degree Atrioventricular Block: A Rare Association

**DOI:** 10.7603/s40602-013-0004-7

**Published:** 2013-10-01

**Authors:** Raymond C.C. Wong, Swee Guan Teo, James W.L. Yip

**Affiliations:** 1 Department of Cardiology, National University Heart Centre Singapore, 1E Kent Ridge Road, Singapore, China; 2 Department of Cardiology, National University Heart Centre Singapore, Singapore, Singapore

A 40 year-old man with no significant past history except for asymptomatic 1^st^ degree heart block presented for general health screen. Clinical examination revealed a loud continuous murmur with diastolic accentuation in the left precordium, noncollapsing pulse, and no differential pulses in the upper and lower limbs. The ECG showed normal sinus rhythm with PR interval of 220 milliseconds.

**Figure 1.**
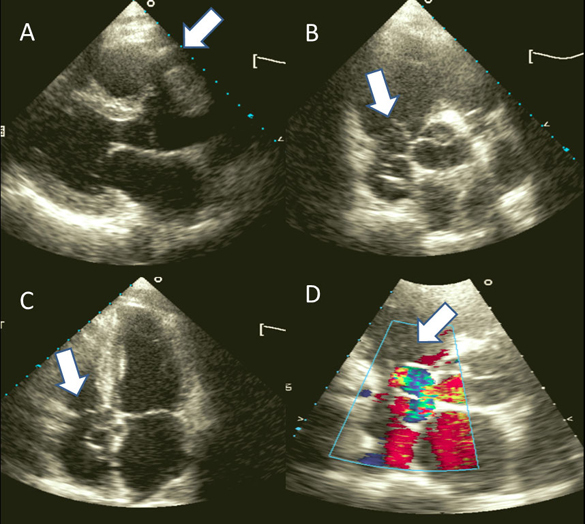
*Transthoracic echocardiogram performed showed the tubular structure coming off the right coronary cusp (bold arrow, image A); saccular formations adjacent to the aortic root in short axis and apical 4 chamber view (bold arrows, image B and C); as well as predominantly diastolic turbulent flow within the saccular formations (image D).*

**Figure 2.**
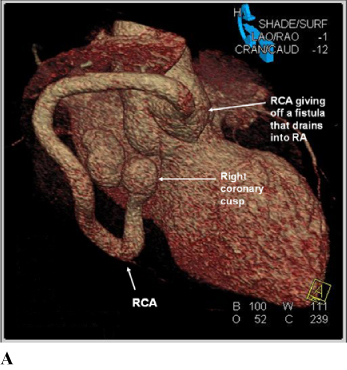

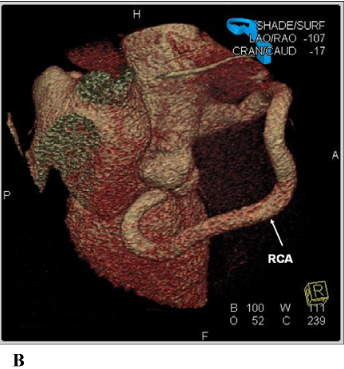

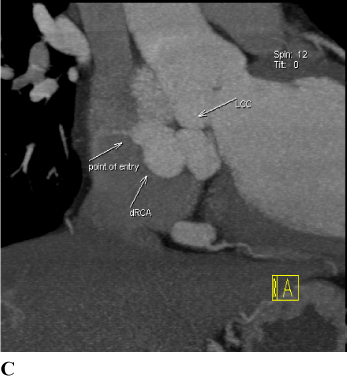

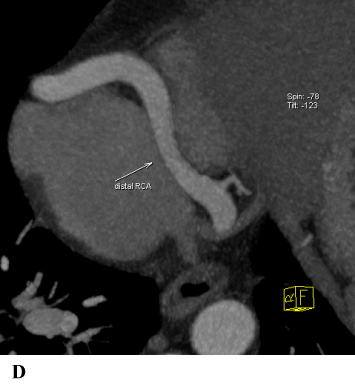
*Cardiac multidetector computed tomography (MDCT) angiogram volume rendering reconstruction of cardiac structures (images A-B) and multiplanar reconstruction images (images C-D) showed a dilated, tortuous right coronary artery (RCA) that eventually gave off a fistulous connection into the right atrium (RA).*

Transthoracic echocardiogram (Figure 1) showed a dilated right coronary artery that was enlarged, forming multiple saccular aneurysms distally that drain into the roof of right atrium. The right heart was normal in size and function. CT angiogram (Figure 2) later confirmed the presence of a coronary artery fistula from the right coronary artery draining into the right atrium. Right coronary artery is dilated and the distal segment of the RCA is tortuous and aneurismal. Nuclear myocardial perfusion imaging showed no inducible ischemia during exercise stress. He declined cardiac catheterization, and was advised for corrective surgery.

This case illustrated a rare saccular aneurysm formation in a right coronary artery to right atrium (coronary-cameral) fistula associated with 1^st^ degree atrioventricular (AV) block. Coronary arteriovenous fistula (CAVF) arises as a persistence of sinusoidal connections between the lumens of the primitive tubular heart in the early embryologic period. It is reported in 0.1%–0.2% of all cases of selective coronary angiography[1]. Most fistulae originate from the right coronary artery (60%) and terminate in the right side of the heart such the right ventricle or atrium, coronary sinus, and pulmonary vasculature. Most often congenital, a CAVF may rarely arise as a consequence of surgical complication, endomyocardial biopsy, invasive procedures[2, 3] or penetrating trauma. It could cause myocardial stealing due to run-off from the coronary vasculature to a low-pressure receiving cavity. Coronary artery that feeds the fistula progressively dilates, leading to complications of frank aneurysmal formation and mural thrombosis. Hence, all CAVF ought to be closed except for trivial ones. The evolution of 1^st^ degree AV block in our case may reflect a flow limitation to atrioventricular (AV) node directly caused by the CAVF.

CT angiogram has now emerged as an excellent imaging technique to delineate the lesion anatomy[4-6] CAVF generally requires closure. Transcatheter embolization techniques using coils, bags, or other devices can be performed successfully and safely, and are now the treatment modality of choice [7]. CAVFs with multiple connections, circuitous routes, and acute angulations may however be better treated surgically.
